# Ten Years of Population-Level Genomic *Escherichia coli* and *Klebsiella pneumoniae* Serotype Surveillance Informs Vaccine Development for Invasive Infections

**DOI:** 10.1093/cid/ciab006

**Published:** 2021-01-07

**Authors:** Samuel Lipworth, Karina-Doris Vihta, Kevin K Chau, James Kavanagh, Timothy Davies, Sophie George, Leanne Barker, Ali Vaughan, Monique Andersson, Katie Jeffery, Sarah Oakley, Marcus Morgan, Timothy E A Peto, Derrick W Crook, A Sarah Walker, Nicole Stoesser

**Affiliations:** 1 Nuffield Department of Medicine, University of Oxford, Oxford, United Kingdom; 2 Oxford University Hospitals NHS Foundation Trust, Oxford, United Kingdom; 3 NIHR Health Protection Research Unit in Healthcare Associated Infections and Antimicrobial Resistance at University of Oxford in partnership with Public Health England, Oxford, United Kingdom; 4 NIHR Biomedical Research Centre, Oxford, United Kingdom

**Keywords:** vaccine, bloodstream infection, antimicrobial resistance, whole genome sequencing, Enterobacteriaceae

## Abstract

**Background:**

The incidence of bloodstream infections (BSIs) caused by *Escherichia coli* and *Klebsiella pneumoniae* is increasing, with substantial associated morbidity, mortality, and antimicrobial resistance. Unbiased serotyping studies to guide vaccine target selection are limited.

**Methods:**

We conducted unselected, population-level genomic surveillance of bloodstream *E. coli* and *Klebsiella pneumoniae* isolates from 2008 to 2018 in Oxfordshire, United Kingdom. We supplemented this with an analysis of publicly available global sequencing data (n = 3678).

**Results:**

We sequenced 3478 *E. coli* isolates (3278 passed quality control) and 556 *K. pneumoniae* isolates (535 [K-antigen] and 549 [O-antigen] passed quality control). The 4 most common *E. coli* O-antigens (O1/O2/O6/O25) were identified in 1499/3278 isolates; the incidence of these O-types increased over time (incidence rate ratio per year [IRRy] = 1.14, 95% confidence interval [CI]: 1.11–1.16). These O-types accounted for 616/1434 multidrug-resistant (MDR) and 173/256 extended-spectrum beta-lactamase (ESBL)-resistant isolates in Oxfordshire but only 19/90 carbapenem-resistant isolates across all studies. For *Klebsiella pneumoniae*, the most common O-antigens (O2v2/O1v1/O3b/O1v2) accounted for 410/549 isolates; the incidence of BSIs caused by these also increased annually (IRRy = 1.09; 95% CI: 1.05–1.12). These O-types accounted for 122/148 MDR and 106/123 ESBL isolates in Oxfordshire and 557/734 carbapenem-resistant isolates across all studies. Conversely we observed substantial capsular antigen diversity. Analysis of 3678 isolates from global studies demonstrated the generalizability of these findings. For *E. coli*, based on serotyping, the ExPEC4V and ExPEC10V vaccines under investigation would cover 46% and 72% of Oxfordshire isolates respectively, and 47% and 71% of MDR isolates.

**Conclusions:**

O-antigen targeted vaccines may be useful in reducing the morbidity, mortality, and antimicrobial resistance associated with *E. coli* and *K. pneumoniae* BSIs.

## Teaser

A large, unselected, 10-year sequencing-based serotype analysis of *Escherichia coli* and *Klebsiella pneumoniae*-associated bloodstream infections (BSIs) (Oxfordshire, UK [n~3827] plus available global sequences [n = 3678]). This strongly supports further development of effective O-antigen-targeted vaccines, which could substantially reduce BSIs and antimicrobial resistance.

The incidence of bloodstream infections (BSIs) caused by Enterobacteriaceae (eg, *Escherichia coli*, *Klebsiella pneumoniae*) continues to increase globally [1–3]. Enterobacteriaceae are a significant antimicrobial resistance (AMR) threat as they can acquire new genes horizontally and therefore rapidly adapt to changing selection pressures, including antimicrobial use. With the limited development of new antimicrobials to treat multidrug-resistant (MDR) infections, the threat of untreatable disease is a significant risk [4]. To date, a nationally mandated set of infection prevention and control standards arising from the Health and Social Care Act of 2008 [5] has had limited success in reducing the incidence of BSIs caused by Enterobacteriaceae, as exemplified by national surveillance data [4, 6].

Prophylactic vaccines represent an alternative approach to combating antimicrobial resistance (AMR) by reducing antibiotic usage and preventing infections caused by AMR-associated strains. For *E. coli*, the O-antigen, a component of lipopolysaccharide (LPS), has been considered the most promising vaccine target [7]. A recently developed bioconjugate vaccine (ExPEC4V; Janssen Pharmaceuticals) targeting 4 *E. coli* O-antigens (O1A, O2, O6A, and O25B) has subsequently shown promise in a phase II study [8]. A phase 1/2 clinical trial for a 10-valent vaccine (ExPEC10V) is currently recruiting [9]. For *K. pneumoniae,* vaccine development is lagging behind [10], and there are no vaccines currently in clinical trials.

For *E. coli*, recent serotyping studies have described limited O-antigen diversity in foodborne [11], enterotoxigenic *E. coli*/ enteropathogenic *E. coli* (ETEC/EPEC) [11], and extended-spectrum beta-lactamase (ESBL)-producing BSI isolates [12]. To our knowledge, however, there are no recent systematic and longitudinal studies characterizing the seroepidemiology of unselected BSIs. Recent studies of multicountry isolate collections, which were nonsystematically collected and/or enriched for antibiotic resistance, have suggested that the O-antigen could also be a promising vaccine target for *K. pneumoniae*. Follador and colleagues analyzed 4 sequencing data sets (1 pre-1950, 2 others enriched for AMR/clonal outbreaks) and found that 3 O-antigens (O1/2/3) accounted for 80% of isolates, whereas the diversity of K-antigens was an order of magnitude higher [13]. Similarly, the O1/2/3/5 antigens accounted for 90.1% of isolates in a recent global sequencing study [14], although there were only relatively small numbers of isolates over limited timeframes from each country, and it was unclear how these had been selected.

Large-scale, systematic typing studies could help to inform the rational selection of vaccine targets across population groups which best mitigate the risk of BSI (including those associated with AMR) and provide a baseline for monitoring the emergence of serotype variation in response to vaccine rollout. We performed a large, systematic study over 10 years in Oxfordshire and combined this with an analysis of publicly available global data sets to provide a robust basis for future vaccine development.

## METHODS

### Sample Collection/Sequencing

All blood culture isolates identified by the Oxford University Hospitals clinical microbiology laboratory between September 2008 and December 2018 as being *K. pneumoniae*/*E. coli* (by matrix-assisted laser desorption/ionization time of flight [MALDI-TOF]) analysis (from 2013) and by growth on chromogenic/MacConkey agar and analytical profile index tests (prior to 2013) were included in the study. One *E. coli* isolate was excluded due to a cataloging error. Infection control procedures were in place for inpatients known to be colonized with ESBL/carbapenemase-producing Enterobacteriaceae (CPE) during the study, including isolation in side rooms where feasible, enhanced contact precautions, and screening of high-risk patients on admission (for CPE). To avoid double counting instances where patients had several positive blood cultures taken during a single episode of infection, we considered only the first available isolate per 90-day period per patient.

Isolates were stored at −80°C in glycerol nutrient broth. Subcultures were grown overnight on Columbia blood agar (Oxoid, Basingstoke, UK) at 37°C. DNA for sequencing was extracted using the QuickGene DNA extraction kit (Autogen, Holliston, Massachusetts, USA) as per the manufacturer’s instructions with the addition of a mechanical lysis step (FastPrep, MP Biomedicals, Irvine, California, USA; 6 m/s for 40 seconds). Sequencing was performed using Illumina HiSeq 2500/3000/4000/MiSeq instruments as previously described [15]. To place our results in a global context, we additionally analyzed 3678 isolates from existing studies selected to encompass the recent national/international longitudinal data sets available in the literature as well as isolates that were relatively rare in our collection (eg, carbapenemase producing/genotypically hypervirulent isolates; data available through NCBI project accession numbers: PRJNA480723 [16], PRJEB4681 [17], PRJEB35000 [18], PRJEB12513 [19], PRJEB30913 [20], PRJEB1271 [21], PRJEB10018 [22, 23]).

### Statistics

Stacked negative binomial regression was used to compare incidence rate ratios over time (per year longer, incidence rate ratio per year [IRRy]) of BSI belonging to different antigen groups [24]. For each serotype or group of serotypes examined, we modeled the incidence of isolates with and without this trait over calendar time. In order to include as many isolates as possible in these models we defined calendar years as beginning in November (ie, isolates before November 2008/after November 2018 are excluded from this part of the analysis). A Wald test for heterogeneity was performed to test the null hypothesis that the incidence trend did not differ between isolates with and without the trait. Healthcare-associated (HA) infection was defined as either nosocomial infection (onset >48 hours after admission) or onset within 30 days of last discharge and community-associated (CA) as that occurring >30 days since last admission [25, 26]. Statistical analysis was performed using R software, version 4.0.2, except for stacked negative binomial regression models, which were implemented in STATA version 15. Sequencing data were linked to electronic healthcare records via the Infections in Oxfordshire Research Database (IORD). IORD has generic Research Ethics Committee, Health Research Authority, and Confidentiality Advisory Group approvals (14/SC/1069, ECC5-017(A)/2009).

### Bioinformatics

De novo assembly was performed using Shovill (version 1.0.4) [27]. In silico serotyping was performed using the Ectyper tool [28–30] for *E. coli* and with Kleborate for *Klebsiella* spp. [31]. For *E. coli* serotypes we included only those isolates that passed the ECtyper quality control (QC) filter and had a high confidence locus call; similarly for *Klebsiella* spp. we included only isolates where the Kleborate locus confidence call was at least “good” (found in a single piece or with ≥95% coverage, with ≤3 missing genes and ≤1 extra genes) [31]. Genes (yersiniabactin, colibactin, and aerobactin) associated with hypervirulence (defined as the ability to cause severe invasive disease in otherwise healthy individuals in the community [31]) were detected using the Kleborate tool. Virulence score was part of the Kleborate output, namely: 0 = no acquired virulence factors, 1 = yersiniabactin only, 2 = yersiniabactin and colibactin (or colibactin only), 3 = aerobactin only, 4 = aerobactin and yersiniabactin, 5 = yersiniabactin, colibactin, and aerobactin.

### Antibiotic Susceptibility Testing

Antibiotic susceptibility was measured using the BD Phoenix (Becton Dickinson, Franklin Lakes, New Jersey, USA) platform (EUCAST breakpoints [32]) for isolates after 2013 or disc diffusion (BSAC breakpoints [33]) for earlier isolates; all susceptibilities were measured using standard operating procedures in a UK Accreditation Service (UKAS) accredited laboratory.

Antibiotic susceptibility data were available through the Infections in Oxfordshire Research Database. For comparisons with existing studies, *in silico* antibiotic resistance gene detection (as called by Kleborate) was used instead of in vitro phenotyping because the latter results were not universally available.

## RESULTS

**Figure 1.**
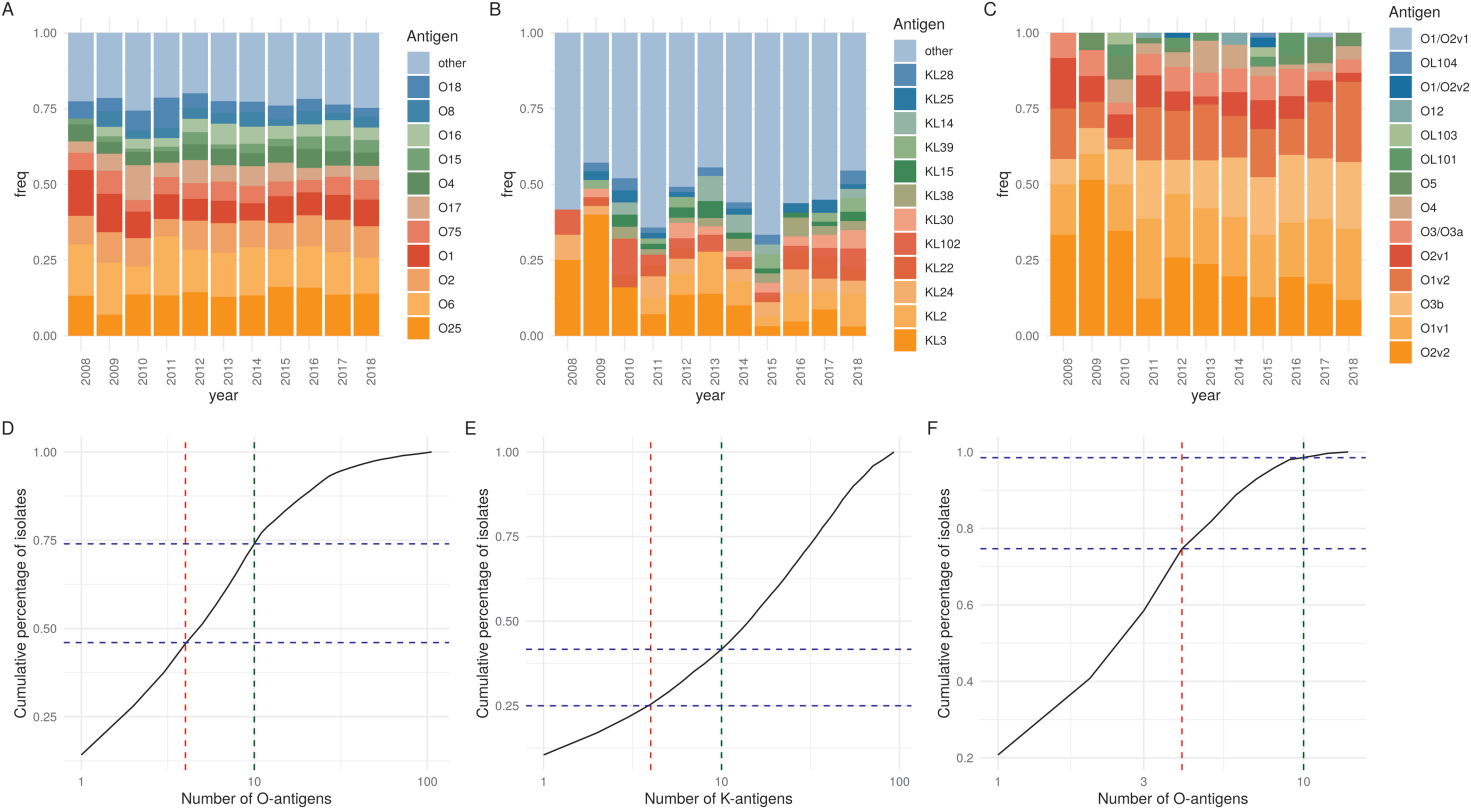
Limited O-antigen diversity in Oxfordshire BSIs with broadly stable population structure over time. Proportion of (*A*) *E. coli* O-antigens, (*B*) *Klebsiella* spp. capsular antigens, and (*C*) *Klebsiella* spp. O-antigens observed in all isolates over the 10-year period. In panels *D*–*F*, red/green dashed lines denote the *x*-axis position of the 4/10 most prevalent O/K-types, and the blue dashed lines demonstrate the cumulative percentage of isolates that have these antigens, for (*D*) *E. coli* O-antigens, (*E*) *Klebsiella* spp. capsular (K) antigens, and (*F*) *Klebsiella* spp. O-antigens. Abbreviation: BSI, bloodstream infection.

A total of 3479 *E. coli* isolates were sequenced of which 3278 passed *in silico* serotyping quality control. From these, 106 unique O-antigens were identified, though a relatively small number of these accounted for the majority of isolates (Figure 1 and Supplementary Table 1). The overall incidence of *E. coli* BSIs increased over the study period IRRy = 1.14; 95% confidence interval [CI]: 1.11–1.16. The 4 most common serotypes (O1A, O2, O6A, and O25B; ie, those covered by the ExPEC4V vaccine) were identified in 1499/3278 (46%) isolates. Their incidence increased over time (IRRy = 1.14; 95% CI: 1.11–1.16); as did non-ExPEC4V serotypes (IRRy = 1.13; 95% CI: 1.10–1.16) (*p*_heterogeneity_ = 0.7, Supplementary Figure 1). There was no evidence that O1/O2/O6/O25 serotypes differed in prevalence between CA and HA isolates (912/1969, 46% vs 576/1292, 45% respectively; *P* = .3) or across 5 age-groups (<18 years 52/122 (43%), 18–39 years 98/222 (44%), 40–59 years 204/477 (43%), 60–79 years 577/1282 (45%), >80 years 557/1158 (48%); *P* = .3). The ExPEC10V serotypes (O1A, O2, O4, O6A, O8, O15, O16, O18A, O25B, and O75) were identified in 2347/3278 (72%) isolates. This vaccine does not include O17, which was the fifth most common O-antigen type in our study, accounting for 181/3278 (6%) isolates.

**Figure 2.**
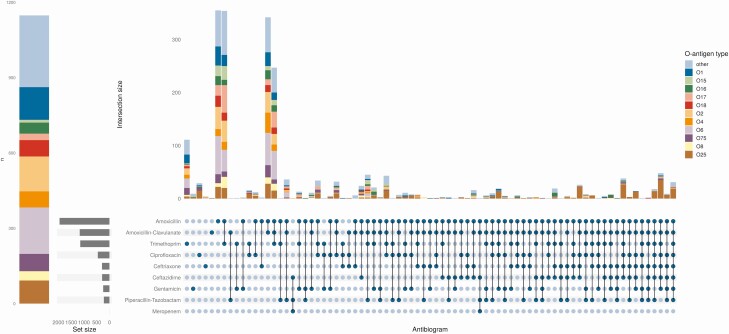
*Left*: Number of fully sensitive (to antibiotics shown in the righthand upset plot) *E. coli* BSI isolates by O-type. *Right*: Upset plot showing the number of *E. coli* isolates with phenotypic resistance to the antibiotics shown, stratified by O-antigen type (colors) and ordered by number of intersections. Horizontal bar plot shows the total number of isolates phenotypically resistance to each antibiotic in any combination. Abbreviation: BSI, bloodstream infection.

Of the 3251 isolates for which in vitro antimicrobial sensitivity data were available, the ExPEC4V serotypes accounted for 191/331 (58%) of ESBL-producing and 394/834 (47%) of multidrug resistant (MDR; resistance to ≥3 antibiotic classes) isolates. The ExPEC10V serotypes accounted for 592/834 (71%) of MDR and 255/331 (77%) of ESBL isolates. O25 was particularly associated with both ESBL (162/450, 36%) and MDR (249/450, 55%) phenotypes (Figure 2). Of the 2 carbapenem-resistant Oxford *E. coli* BSI isolates, neither had an O-antigen contained in the ExPEC4V/10V vaccines (one was O17 and the other O9).

Similarly we sequenced 556 *K. pneumoniae* (and *quasipneumoniae*) isolates; 93 unique K- and 14 unique O-loci were confidently identified in 535 (K-antigen)/549 (O-antigen) isolates passing quality control (Supplementary Tables 2 and 3). The incidence of *K. pneumoniae* BSIs also increased overall over the study period IRRy = 1.09; 95% CI: 1.05–1.12. To determine the theoretical feasibility of developing a *K. pneumoniae* vaccine and for comparison with *E. coli*, we again considered the top 4 K- and O-loci. KL2, KL22, KL24, and KL3 were identified in 136/535 (25%) isolates (Figure 1). There was no evidence that the incidence of BSIs caused by this group of capsular types changed over time (IRRy = 1.03; 95% CI: .94–1.13), nor that incidence trends varied between these and other *Klebsiella* spp. BSIs (*p*_heterogeneity_ = 0.23). By contrast, O2v2, O1v1, O3b, and O1v2 accounted for 410/549 (75%) isolates and the incidence of BSIs caused by these increased over time (IRRy = 1.09; 95% CI: 1.05–1.12), although again there was no evidence that these incidence trends differed from all other O-types (*p*_heterogeneity_ = 0.8). A comparable *Klebsiella* vaccine to the ExPEC10V (ie, composed of the 10 most common O-antigens, O2v2, O1v1, O3b, O1v2, O2v1, O3/O3a, O4, O5, OL101, and 1 of O1/O2v2, O12, OL103 [which were tied for 10th most common O-antigen]), would cover 541/549 (99%) of isolates in our study.

KL3 and KL30 were the most common capsular antigens associated with the ESBL phenotype (43/123 [35%] and 12/123 [10%], respectively). KL3 was also the K-antigen most commonly associated with MDR (45/148 [30%]). In total, the 4 most common K-antigens overall (KL2/242/24/3) accounted for 50/123 (41%) of ESBL and 65/148 (44%) of MDR isolates. In contrast, the 4 most common O-antigens accounted for 106/123 (86%) of ESBL and 122/148 (82%) MDR isolates, with no evidence that the proportion of HA/CA BSI caused by these 4 antigens differed (179/238 [75%] vs 230/310 [74%], respectively; *P* = .9).

**Figure 3.**
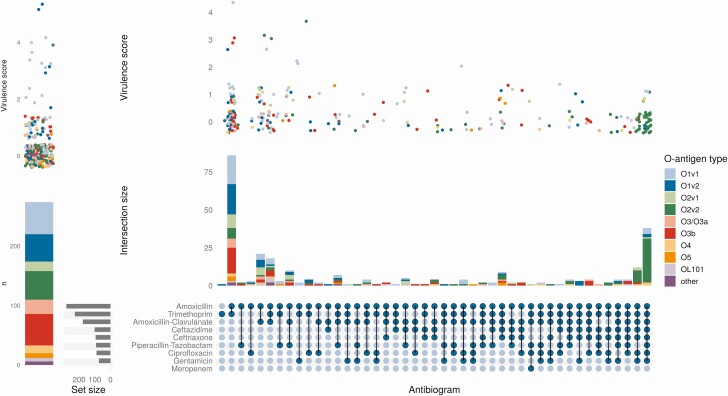
*Left*: Number of *K. pneumoniae* BSI isolates either fully susceptible or resistant only to amoxicillin shown by O-antigen type. The jitter plot above shows the distribution of virulence scores, points are colored according to their O-antigen type. *Right*: Upset plot showing the number of *K. pneumoniae* isolates with resistance to the classes of antibiotics shown. Stacked bar plot shows the distribution of these by O-antigen type, and the jitter plots above demonstrate the corresponding distributions of virulence scores. Virulence score is as defined by Kleborate (see methods). Abbreviation: BSI, bloodstream infection.

Previous studies have suggested that clinically hypervirulent *K. pneumoniae* isolates (ie, causing CA invasive infection) are particularly associated with the KL1/2 capsular types [34]. There were only 2 isolates with the KL1 capsular antigen in our study, only 1 of which carried the virulence-associated genes encoding for yersiniabactin, colibactin, aerobactin, salmochelin, and *rmpA*. Although KL2 did have the most isolates carrying hypervirulence genes of any capsular type, there were also 13/35 KL2 isolates with no hypervirulence loci (Figure 3). Also, 36/37 KL1/2 isolates had O1v1 or O1v2 antigens (the other had O1/O2v2) meaning that a quadrivalent O-antigen-targeted vaccine would theoretically provide good coverage against this important group of isolates. Although there were more CA than HA isolates for the KL2 capsular type (19/15), this was not the only K-type where this was true, and the clinically hypervirulent phenotype was observed in 74 capsular types in total.

As the isolates from our study originated from a single region of the United Kingdom with a relatively low AMR burden, we compared our serotypes to those from recently published studies in the United Kingdom [17, 19, 21] and globally [16, 18, 20, 22, 23]. Overall, although there was some regional variation, findings supported the potential for O-antigen-targeted quadrivalent vaccines to contribute to substantial reductions in overall BSIs, as well as those caused by ESBL and MDR isolates, in both *E. coli* and *K. pneumoniae* (Table 1). For *K. pneumoniae* they would likely provide a good level of protection against carbapenem-resistant and (genetically) hypervirulent isolates.

**Table 1.** Comparison of the Oxford BSIs With Those From Similar National/Regional Studies

*E. coli* - O antigen	Proportion from O1/O2/O6/O25						
Study	Oxford	CUH [10, 14]	EUSCAPE [15, 16]	SE Asia [13]	Netherlands [11]	Australia [9]	Scotland [12]
Isolate group							
All	1499/3278 (46%)	205/399 (51%)	130/329 (40%)	NA	188/445 (42%)	44/79 (56%)	64/159 (40%)
ESBL	173/256 (68%)	30/36 (83%)	61/154 (40%)	NA	113/244 (46%)	12/15 (80%)	12/15 (80%)
MDR	616/1434 (43%)	95/192 (49%)	87/244 (36%)	NA	113/274 (41%)	26/44 (59%)	27/85 (32%)
Carbapenem resistant	0/2 (0%)	NA	18/86 (21%)	NA	1/2 (50%)	NA	NA
*K. pneumoniae* - K antigen	Proportion from KL2/ KL242/KL24/KL3						
All	136/535 (25%)	21/152 (14%)	261/1634 (16%)	56/243 (23%)	NA	NA	NA
ESBL	50/123 (41%)	8/94 (9%)	124/856 (14%)	30/107 (28%)	NA	NA	NA
MDR	65/148 (44%)	12/110 (11%)	181/1221 (15%)	37/130 (28%)	NA	NA	NA
Carbapenem resistant	1/3 (33%)	0/2 (0%)	81/678 (12%)	2/15 (13%)	NA	NA	NA
Hypervirulent	15/28 (54%)	0/1 (0%)	21/55 (38%)	20/72 (28%)	NA	NA	NA
*K. pneumoniae* - O antigen	Proportion from O2v2, O1v1, O3b and O1v2						
All	410/549 (75%)	119/154 (77%)	1173/1651 (71%)	228/318 (72%)	NA	NA	NA
ESBL	106/123 (86%)	84/96 (88%)	592/864 (68%)	116/157 (74%)	NA	NA	NA
MDR	122/148 (82%)	98/112 (88%)	884/1231 (72%)	137/191 (72%)	NA	NA	NA
Carbapenem resistant	0/3 (0%)	2/2 (100%)	521/684 (76%)	34/45 (76%)	NA	NA	NA
Hypervirulent	27/28 (96%)	1/1 (100%)	47/55 (85%)	70/87 (80%)	NA	NA	NA

For each group we considered the proportion of isolates in each study carrying the 4 most prevalent antigens in the Oxford Data Set to consider the hypothetical coverage a vaccine targeted at these might provide. In total, 3479 *E. coli* isolates from Oxford were sequenced of which 3278 passed Ectyper QC. And 556 Oxford *K. pneumoniae* isolates were sequenced of which 535 (K-antigen)/549 (O-antigen) passed Kleborate QC; 1510 *E. coli* isolates from published data sets were included, of which 1411 passed Ectyper QC. Of the 2168 *K. pneumoniae* isolates included from published data sets, 2029 (K-antigen)/2123 (O-antigen) passed Kleborate QC typing. ESBL/MDR/carbapenem resistant numbers are based on gene detection from Kleborate, including for Oxford isolates (in contrast to the main text where we report the results of in vitro drug susceptibility testing).

Abbreviations: BSI, bloodstream infection; CUH, Cambridge University Hospitals; ESBL, extended-spectrum beta-lactamase; EUSCAPE, European Survey of Carbapenemase-Producing Enterobacteriaceae; MDR, multidrug resistant; NA, not applicable; QC, quality control; SE Asia, Southeast Asia.

Conversely for *E. coli*, only 19/90 (21%) isolates carrying carbapenem resistance genes in all studies carried O-antigens covered by the ExPEC4V vaccine, with the O102 (n = 18), O25 (n = 11), O8 (n = 11) and O160 (n = 10) antigens predominating in these isolates. This was only slightly improved when considering the antigens in the ExPEC10V vaccine, which would cover 35/90 (39%) isolates in all studies. Reflecting this, only 13/90 isolates carrying carbapenem resistance genes belonged to the major group of sequence types (STs) (131/95/73/69), which are responsible for most (predominantly CA) *E. coli* BSIs reported previously [16, 17, 19].

## DISCUSSION

A quadrivalent vaccine comprising the 4 most prevalent O-antigens for *E. coli* and *Klebsiella* spp. could theoretically have a significant impact on the incidence of BSIs and ESBL/MDR infections, as well as offering an alternative approach to help tackle the global epidemic of AMR in Enterobacteriaceae. Ten-valent vaccines are at a slightly earlier stage of development for *E. coli,* but our data suggest that they have the potential to make a significant impact on the incidence of invasive disease and associated antimicrobial resistance. Such a vaccine for *K. pneumoniae* would have covered nearly all isolates (541/549 [99%]) associated with invasive disease over the past decade in Oxfordshire.

Vaccines should also provide some protection against non-BSI infections that may evolve into BSIs, in particular urinary tract infections. As well as reducing morbidity and providing economic benefits, this could also be expected to lower antibiotic selection pressures against *E. coli* and *Klebsiella* spp. The relatively much higher incidence of these primary infections versus BSIs may increase the economic viability of any prospective vaccine that, particularly for *Klebsiella* spp., may be a significant barrier to development given the difficulty in identifying groups of patients in which vaccination would be cost-effective [35].

We would expect the ExPEC4V/ExPEC10V vaccines to offer less protection against carbapenem-resistant *E. coli* infections. Although at first glance this looks disappointing, the prevention of ESBL infections would still reduce the carbapenem selection pressure. Additionally, it was notable that the majority of carbapenem resistance genes carrying *E. coli* isolates were not from the sequence types that have been found to cause most (predominantly CA) BSIs in other studies [16, 17, 19]. This might indicate that these isolates do not originate from the community reservoir and that they may be associated with nosocomial transmission as was found to be the case in *K. pneumoniae* [23]. Widespread dissemination of carbapenem resistance in sequence types associated with global epidemics of CA-MDR disease (eg, ST131) would be a public health challenge, and a vaccine may have a role in preventing this.

The main limitation of our study is that it is from a single region. To mitigate this we analyzed data from other published studies; however, in contrast to our long-term regional surveillance, most of these were either deliberately enriched for MDR isolates or at high risk of bias due to unclear sampling methodologies. Although direct comparison with our data is therefore difficult, the overall findings appear to be reflected both nationally and globally. We were unable to characterize the distribution of capsular (K) antigen types for *E. coli* because, to our knowledge, there is no curated database of sequences currently available. Additionally, we were unable to assign an in silico O-antigen call to ~5% of isolates in both *E. coli* and *K. pneumoniae*. This may be due to assembly errors or the presence of novel serotypes.

Overall, O-antigens targeted vaccines for *E. coli* and *Klebsiella* spp. could theoretically have a significant impact on the incidence of BSIs and ESBL/MDR infections, as well as offering an alternative approach to help tackle the global epidemic of AMR in Enterobacteriaceae. Our findings support future development and subsequent efficacy studies for such vaccines, provided that they are demonstrated to be safe and immunogenic. The substantial capsular antigen diversity observed for *Klebsiella* spp. BSIs would likely preclude this as a useful vaccine target. Surveillance following the implementation of any vaccine would be essential in identifying any evidence of strain replacement.

## Supplementary Data

Supplementary materials are available at *Clinical Infectious Diseases* online. Consisting of data provided by the authors to benefit the reader, the posted materials are not copyedited and are the sole responsibility of the authors, so questions or comments should be addressed to the corresponding author.

ciab006_suppl_Supplementary_Materials

## Notes

**Author contributions.** S. L., A. S. W., and N. S. conceived the study. Sample collection, DNA extraction, and sequencing were performed by J. K., K. K. C., A. V., K. J., M. A., T. D., L. B., S. G., S. O., N. S., and M. M. S. L. performed the analysis of the sequencing data. S. L., N. S., and A. S. W. wrote the manuscript. T. E. A. P., A. S. W., N. S., and D. W. C. provided supervision, funding, and resources. All authors read and commented on the manuscript. All authors read and approved the final manuscript.

***Acknowledgments.*** This work uses data provided by patients and collected by the UK’s National Health Service as part of their care and support. We thank all the people of Oxfordshire who contribute to the Infections in Oxfordshire Research Database.

Research Database Team: L Butcher, H Boseley, C Crichton, DW Crook, D Eyre, O Freeman, J Gearing (community), R Harrington, K Jeffery, M Landray, A Pal, TEA Peto, TP Quan, J Robinson (community), J Sellors, B Shine, AS Walker, D Waller. Patient and Public Panel: G Blower, C Mancey, P McLoughlin, B Nichols. We would like to thank the EUSCAPE consortium for making their data available in the public domain. We are also grateful for the efforts of the many biomedical and research scientists, in particular, Dai Griffiths, who have assisted this project over the past 10 years. All sequencing data created for this study has been deposited in the NCBI under study accession number PRJNA604975. IORD has generic Research Ethics Committee, Health Research Authority and Confidentiality Advisory Group approvals (14/SC/1069, ECC5-017(A)/2009).

***Financial support.*** The research was supported by the National Institute for Health Research (NIHR) Health Protection Research Unit in Healthcare Associated Infections and Antimicrobial Resistance (grant number NIHR200915) at the University of Oxford in partnership with Public Health England (PHE) and by Oxford NIHR Biomedical Research Centre. T. E. A. P. and A. S. W. are NIHR senior investigators. The report presents independent research funded by NIHR. The views expressed in this publication are those of the authors and not necessarily those of the NHS, NIHR, the Department of Health, or Public Health England. The computational aspects of this research were funded from the NIHR Oxford BRC with additional support from the Wellcome Trust Core Award grant number 203141/Z/16/Z. S. L. is supported by a Medical Research Council Clinical Research Training Fellowship. K. C. is funded by the Medical Research Foundation.

***Potential conflicts of interest.*** All authors: No reported conflicts of interest. All authors have submitted the ICMJE Form for Disclosure of Potential Conflicts of Interest. Conflicts that the editors consider relevant to the content of the manuscript have been disclosed.
