# Supplementary material for: Ten Years of Population-Level Genomic Escherichia coli and Klebsiella pneumoniae Serotype Surveillance Informs Vaccine Development for Invasive Infections
Source: Clin Infect Dis. 2021 Jan 7;73(12):2276–82. doi: 10.1093/cid/ciab006 (PMC8677521; doi:10.1093/cid/ciab006)
Supplement: ciab006_suppl_Supplementary_Materials [file ciab006_suppl_supplementary_materials.docx]

**Supplementary Appendix**


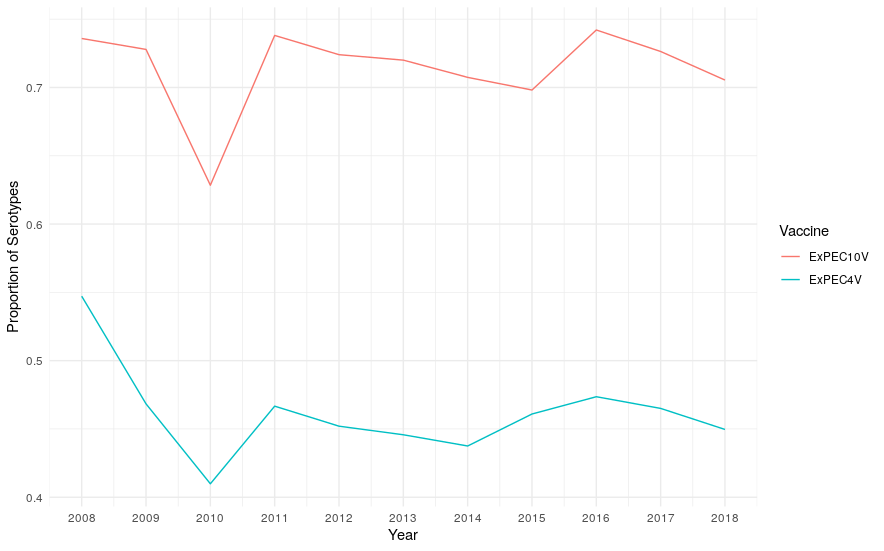


**Figure S1**: Proportion of *E. coli* bloodstream infection serotypes covered by the ExPEC10V/4V vaccines over time

| **MLST** | **O-type** | **n** |
| --- | --- | --- |
| 12 | O18 | 14 |
| 12 | O4 | 128 |
| 12 | other | 1 |
| 127 | O15 | 1 |
| 127 | O6 | 108 |
| 131 | O1 | 1 |
| 131 | O15 | 1 |
| 131 | O16 | 91 |
| 131 | O25 | 356 |
| 131 | O75 | 1 |
| 131 | other | 2 |
| 69 | O15 | 81 |
| 69 | O25 | 9 |
| 69 | other | 217 |
| 73 | O18 | 51 |
| 73 | O2 | 93 |
| 73 | O25 | 45 |
| 73 | O6 | 336 |
| 73 | other | 30 |
| 95 | O1 | 154 |
| 95 | O18 | 27 |
| 95 | O2 | 97 |
| 95 | O25 | 13 |
| 95 | O6 | 1 |
| 95 | other | 21 |
| other | O1 | 113 |
| other | O15 | 20 |
| other | O16 | 58 |
| other | O18 | 50 |
| other | O2 | 121 |
| other | O25 | 32 |
| other | O4 | 34 |
| other | O6 | 20 |
| other | O75 | 179 |
| other | O8 | 112 |
| other | other | 661 |

**Table S1**: distribution of O-types by MLST in *E. coli*. MLSTs with > 100 isolates and the O-antigens in the ExPEC10V vaccine are shown, all others are classified as ‘other’.

| **MLST** | **K-locus** | **N** |
| --- | --- | --- |
| other | KL10 | 10 |
| other | KL102 | 17 |
| other | KL14 | 10 |
| other | KL15 | 8 |
| other | KL2 | 23 |
| other | KL22 | 19 |
| other | KL24 | 12 |
| other | KL25 | 4 |
| other | KL27 | 10 |
| other | KL28 | 3 |
| other | KL3 | 11 |
| other | KL30 | 12 |
| other | KL38 | 9 |
| other | KL39 | 12 |
| other | KL54 | 5 |
| other | KL62 | 6 |
| other | other | 230 |
| ST14 | KL2 | 12 |
| ST14 | other | 5 |
| ST17 | KL25 | 7 |
| ST17 | KL62 | 1 |
| ST17 | other | 2 |
| ST20 | KL102 | 1 |
| ST20 | KL22 | 1 |
| ST20 | KL28 | 8 |
| ST20 | KL38 | 1 |
| ST20 | other | 1 |
| ST29 | KL30 | 5 |
| ST29 | KL54 | 5 |
| ST29 | other | 3 |
| ST37 | KL102 | 1 |
| ST37 | KL14 | 1 |
| ST37 | KL15 | 4 |
| ST37 | KL38 | 6 |
| ST37 | other | 6 |
| ST45 | KL24 | 13 |
| ST45 | KL62 | 3 |
| ST45 | other | 3 |
| ST490 | KL3 | 45 |

​

**Table S2**: Distribution of K-locus types by MLSTs in *K. pneumoniae*. Only MLSTs occurring >10 times and the top 10 (and ties) K-loci in the dataset are shown, others are classified as ‘other’.

| **ST** | **O-locus** | **N** |
| --- | --- | --- |
| other | O1/O2v2 | 2 |
| other | O12 | 3 |
| other | O1v1 | 82 |
| other | O1v2 | 70 |
| other | O2v1 | 22 |
| other | O2v2 | 59 |
| other | O3/O3a | 35 |
| other | O3b | 87 |
| other | O4 | 19 |
| other | O5 | 9 |
| other | OL101 | 10 |
| other | OL103 | 2 |
| other | other | 2 |
| ST14 | O1v1 | 17 |
| ST17 | O2v2 | 2 |
| ST17 | O5 | 7 |
| ST17 | OL101 | 1 |
| ST20 | O1v1 | 1 |
| ST20 | O1v2 | 7 |
| ST20 | O2v2 | 4 |
| ST20 | O3b | 1 |
| ST29 | O1/O2v2 | 1 |
| ST29 | O1v2 | 12 |
| ST35 | O1v1 | 10 |
| ST35 | O2v1 | 1 |
| ST37 | O2v1 | 1 |
| ST37 | O2v2 | 4 |
| ST37 | O3/O3a | 1 |
| ST37 | O3b | 8 |
| ST37 | O4 | 4 |
| ST37 | OL103 | 1 |
| ST45 | O2v1 | 16 |
| ST45 | O3/O3a | 1 |
| ST45 | O3b | 1 |
| ST45 | OL101 | 1 |
| ST490 | O2v2 | 45 |

​

**Table S3:** Distribution of O-locus types by MLSTs in *K. pneumoniae*. Only MLSTs occurring >10 times and the top 10 (and ties) O-loci in the dataset are shown, others are classified as ‘other’.
